# Novel hypophysiotropic AgRP2 neurons and pineal cells revealed by BAC transgenesis in zebrafish

**DOI:** 10.1038/srep44777

**Published:** 2017-03-20

**Authors:** Inbal Shainer, Adi Buchshtab, Thomas A. Hawkins, Stephen W. Wilson, Roger D. Cone, Yoav Gothilf

**Affiliations:** 1 Department of Neurobiology, George S. Wise Faculty of Life Sciences, Tel-Aviv University, Tel-Aviv, Israel; 2 The Department of Cell and Developmental Biology, Faculty of Life Sciences, University College London, London, UK; 3 Life Sciences Institute, University of Michigan, Ann Arbor, MI, USA; 4 Sagol School of Neuroscience, Tel-Aviv University, Tel-Aviv, Israel

## Abstract

The neuropeptide agouti-related protein (AgRP) is expressed in the arcuate nucleus of the mammalian hypothalamus and plays a key role in regulating food consumption and energy homeostasis. Fish express two *agrp* genes in the brain: *agrp1*, considered functionally homologous with the mammalian AgRP, and *agrp2*. The role of *agrp2* and its relationship to *agrp1* are not fully understood. Utilizing BAC transgenesis, we generated transgenic zebrafish in which *agrp1*- and *agrp2*-expressing cells can be visualized and manipulated. By characterizing these transgenic lines, we showed that *agrp1*-expressing neurons are located in the ventral periventricular hypothalamus (the equivalent of the mammalian arcuate nucleus), projecting throughout the hypothalamus and towards the preoptic area. The *agrp2* gene was expressed in the pineal gland in a previously uncharacterized subgroup of cells. Additionally, *agrp2* was expressed in a small group of neurons in the preoptic area that project directly towards the pituitary and form an interface with the pituitary vasculature, suggesting that preoptic AgRP2 neurons are hypophysiotropic. We showed that direct synaptic connection can exist between AgRP1 and AgRP2 neurons in the hypothalamus, suggesting communication and coordination between AgRP1 and AgRP2 neurons and, therefore, probably also between the processes they regulate.

The vertebrate neuro-endocrine system regulates an array of homeostatic processes, allowing the organism to constantly adapt to its surroundings and to the changing availability of resources. Energy homeostasis is regulated by a conserved hypothalamic system. A particularly well-characterized component of this system includes two types of neurons with opposing effects: appetite-stimulating neurons that express agouti-related protein (AgRP), and appetite-inhibiting neurons that express α-melanocyte-stimulating hormone (α-MSH), a derivative of the polypeptide precursor pro-opiomelanocortin (POMC)^1^,^2^. The inhibitory effect of hypothalamic α-MSH on food intake and energy storage is mediated through the melanocortin-4 receptor (MC4R), one of five melanocortin receptors^1^. α-MSH also has a peripheral role in regulating pigmentation through melanocortin-1 receptor (MC1R)^3^,^4^,^5^. Hypothalamic AgRP acts as an appetite stimulator by blocking MC4R signaling, thereby preventing α-MSH-induced inhibition of food intake; it is also an inverse agonist, inhibiting the constitutive activity of MC4R^6^,^7^. Intracerebroventricular (ICV) administration of AgRP or of a synthetic inverse MC4R agonist leads to increased food intake^8^,^9^. Likewise, ectopic overexpression of AgRP in transgenic mice leads to increased food consumption and body weight^6^. Activation of AgRP neurons is sufficient to induce feeding behavior^10^, while their ablation leads to acute starvation^11^.

The role of AgRP in feeding behavior is conserved among evolutionarily distinct vertebrates. Expression of *agrp* mRNA is dramatically upregulated in the hypothalamus in fasting zebrafish^12^ and goldfish^13^, as is the case in mammals^1^,^7^. Administration of MC4R agonists inhibits food intake in goldfish and rainbow trout, whereas ICV injection of synthetic MC4R inverse agonists stimulates food intake in these species^13^,^14^,^15^. Moreover, ectopic overexpression of AgRP in transgenic zebrafish increases body weight and length in the adult^16^. Teleost fish, such as the zebrafish, possess two *agrp* genes, *agrp1* and *agrp2*^17^,^18^,^19^,^20^,^21^, possibly as a result of a whole-genome duplication event^22^,^23^,^24^,^25^,^26^. Tissue distribution analysis of *agrp1* and *agrp2* transcripts in several teleost fish species, mainly by RT-PCR, has indicated that both genes are expressed in the brain, as well as in variety of peripheral tissues^13^,^17^,^18^,^19^,^20^. In the zebrafish, *agrp1* is expressed exclusively in the hypothalamus, and, like mammalian *Agrp*, was shown to be involved in feeding behavior^12^,^27^. Zebrafish *agrp2* mRNA is expressed in the pineal gland^28^,^29^,^30^ and was suggested, on the basis of morpholino-mediated knock-down experiments, to regulate background pigment adaptation for camouflage^30^. Pineal gland expression of *agrp2* was reported in sea bass (*Dicentrarchus labrax*)^20^, and we have also detected its expression in the pineal gland of Nile tilapia (*Oreochromis niloticus*) and rainbow trout (*Oncorhynchus mykiss*; unpublished data). This suggests a conserved pineal gland expression of *agrp2* among teleosts. However, the targets and role of AgRP2 and its relationship to AgRP1 are not fully understood.

To facilitate elucidation of the functions of AgRP1 and AgRP2 in zebrafish, we generated transgenic fish in which *agrp1*- and *agrp2*-expressing cells are fluorescently labelled. Here we describe the anatomical organization of these cells and provide tools for their manipulation. Focusing mainly on AgRP2, we report previously uncharacterized *agrp2*-expressing pineal cells, novel hypophysiotropic AgRP2 neurons, and a novel AgRP1-AgRP2 neuronal interaction. Together, these observations and use of the novel transgenic fish lines may eventually lead to identification of hitherto undiscovered neuroendocrine functions for AgRP neuropeptides.

## Results

### Generation of transgenic zebrafish lines for visualization of AgRP1 and AgRP2 neurons

To visualize AgRP1 and AgRP2 neurons we employed the bacterial artificial chromosome (BAC) transgenesis approach to express fluorescent markers within these neurons. To increase the utility of the generated transgenic lines, we employed the Gal4-VP16 transactivation system, generating BACs in which Gal4-VP16 is under the control of *agrp1* and *agrp2* regulatory regions. BAC clones containing AgRP1- or AgRP2-coding sequences, as well as 45−60 kb downstream and upstream flanking regions, were modified by recombineering according to an established protocol^31^ and used for transgenesis (Supplementary Fig. S1). These BACs are likely to contain all of the *cis*-regulatory elements needed to replicate the endogenous spatial and temporal expression of *agrp1* and *agrp2*. The generated transgenic lines were registered in the Zebrafish Model Organism Database (ZFIN) as TgBAC(*agrp*:Gal4-VP16)^tlv04^ and TgBAC(*agrp2*:Gal4-VP16)^tlv05^. To fluorescently label the Gal4-VP16-expressing cells, we crossed these lines with Tg(UAS:nfsB-mCherry)^c264^ fish^32^. For simplicity, the resulting double-transgenic fish, TgBAC(*agrp*:Gal4-VP16)^tlv04^;Tg(UAS:nfsB-mCherry)^c264^ and TgBAC(*agrp2*:Gal4-VP16)^tlv05^;Tg(UAS:nfsB-mCherry)^c264^, will be referred to as *agrp1*:mCherry and *agrp2*:mCherry, respectively. In addition, we generated a BAC transgenic line expressing enhanced green fluorescent protein (EGFP) under *agrp2* regulatory regions, registered in ZFIN as TgBAC(*agrp2*:EGFP)^tlv06^. For simplicity, these transgenic fish will be referred to as *agrp2*:EGFP.

**Figure 1:**
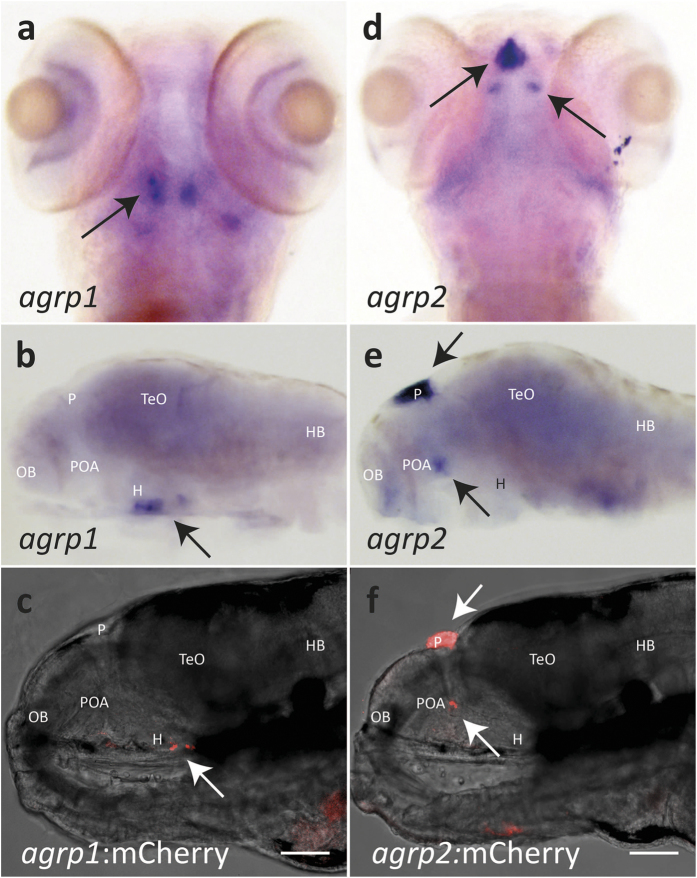
AgRP1 and AgRP2 BAC transgenic lines reflect endogenous *agrp1* and *agrp2* expression patterns. Endogenous mRNA expression of *agrp1* and *agrp2* was compared to the transgene expression in *agrp1*:mCherry and *agrp2*:mCherry larvae, respectively, at 6 dpf. (**a**,**b**) ISH analysis for *agrp1* mRNA expression in a wild-type larva at 6 dpf. (**a**) Ventral and (**b**) lateral views of larvae brains. *agrp1* mRNA expression is localized to the ventral periventricular hypothalamus. (**c**) Lateral view of a 6-dpf *agrp1*:mCherry transgenic larva. Specific mCherry signal is observed in the ventral periventricular hypothalamus (arrow), which replicates the expression pattern of *agrp1* mRNA. (**d**,**e**) ISH analysis of *agrp2* mRNA expression in a 6-dpf wild-type larva. (**d**) Dorsal and (**e**) lateral views of larvae brains. Strong expression of *agrp2* mRNA is observed in the pineal gland (top arrow); weaker bilateral *agrp2* mRNA expression is observed in the preoptic area (bottom arrow). (**f**) Lateral view of the brain of a 6-dpf *agrp2*:mCherry transgenic larva. The expression pattern of mCherry replicates both pineal (top arrow) and preoptic (bottom arrow) *agrp2* mRNA expression. (**a**,**d**) Anterior to top; (**b**,**c**,**e**,**f**) anterior to left. H, hypothalamus; HB, hindbrain; OB, olfactory bulb; P, pineal gland; POA, preoptic area; TeO, optic tectum. Scale bar, 100 μm.

Whole mount *in-situ* hybridization (ISH) of *agrp1* and *agrp2* mRNAs in non-transgenic wild-type larvae was used to determine whether the transgene expression patterns accurately report the endogenous expression of *agrp1* and *agrp*2. Endogenous *agrp1* mRNA expression was found to be restricted to the ventral periventricular hypothalamus (Fig. 1a,b), as previously described^12^. This expression pattern was similar to the distribution of fluorescently labelled cells in *agrp1*:mCherry larvae (Fig. 1c). Whole-mount ISH showed that *agrp2* mRNA is strongly expressed in the pineal gland, as previously described^28^,^29^,^30^. In addition, a domain with weaker *agrp2* expression was revealed in the preoptic area (Fig. 1d,e). The spatial expression of *agrp2* mRNA was matched by expression of the transgene in *agrp2*:mCherry and *agrp2*:EGFP larvae (Fig. 1f). Both lines showed strong fluorescence in the pineal gland, with a weaker fluorescent signal in the preoptic area.

The expression pattern of fluorescent markers in those transgenic lines also correlated with the temporal expression of the endogenous genes. *agrp1* mRNA was detected as early as 2 days post-fertilization (dpf) as previously described^12^, and mCherry expression in the *agrp1*:mCherry line was detected by 3 dpf. *agrp2* mRNA was detected in the pineal at 30 hours post-fertilization and in the preoptic area at 2−3 dpf. mCherry expression in the transgenic line was detected at the same developmental stages, while EGFP expression in the *agrp2*:EGFP line was weaker and appeared later (data not shown).

Together, the above findings indicated that the generated transgenic lines reliably report the endogenous expression of *agrp1* and *agrp2*, and can therefore be used to examine AgRP1 and AgRP2 neuronal systems. For the remainder of this study we focused mainly on the two populations of AgRP2 cells in the pineal and preoptic areas of the forebrain, and also on potential connectivity between AgRP1 and AgRP2 neurons.

### *agrp2* is expressed in a subset of uncharacterized pineal cells

**Figure 2:**
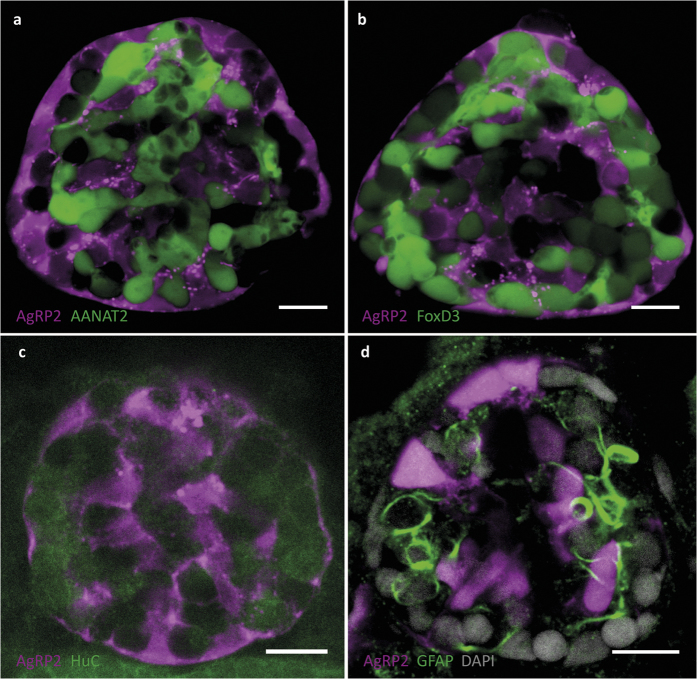
AgRP2 is expressed in uncharacterized pineal cells. (**a**) Pineal gland of a 5-dpf *agrp2*:mCherry, Tg(*aanat2*:EGFP)^y9^ larva. AgRP2 cells (magenta) do not co-localize with pineal photoreceptor cells (green). (**b**) Pineal gland of a 5-dpf *agrp2*:mCherry, Tg(*foxd3*:EGFP)^zf104^ larva. AgRP2 cells (magenta) do not co-localize with *FoxD3* pineal neurons (green). (**c**) Double immunostaining of a 5-dpf *agrp2*:mCherry larva with an antibody against HuC (a neuronal marker) and with anti-RFP. AgRP2 cells (magenta) do not co-localize with HuC-positive cells (green). (**d**) Immunostaining of a 5-dpf *agrp2*:EGFP larva with an antibody against GFAP (a marker for pineal interstitial cells) and with anti-EGFP. AgRP2 cells (magenta) do not co-localize with pineal interstitial cells (green). Scale bar, 10 μm.

The pineal gland of teleost fish contains at least three main types of cells: photoreceptor cells, projection neurons, and interstitial cells^33^,^34^,^35^,^36^. We used transgenic and immunohistochemical approaches to determine the identity of the AgRP2 cells. Pineal photoreceptor cells express opsins and melatonin-synthesizing enzymes such as arylalkylamine *N*-acetyltransferase2 (AANAT2), and are fluorescently labelled in Tg(*aanat2*:EGFP)^y9^ fish^37^. Foxd3 is an early marker of pre-migratory neural crest cells and of certain non-crest-derived cell types including pineal projecting neurons; the latter are fluorescently labelled in Tg(*foxd3*:EGFP)^zf104^ fish^38^,^39^,^40^. We crossed *agrp2*:mCherry with either Tg(*aanat2*:EGFP)^y9^ or Tg(*foxd3*:EGFP)^zf104^ fish. Examination of the resulting double-labelled progeny revealed that the expression of mCherry in AgRP2 cells does not co-localize with EGFP-expressing cells in either line, indicating that they are not likely to be pineal photoreceptors or *foxd3*-expressing pineal neurons (Fig. 2a,b, Supplementary Movie S1). To further examine whether AgRP2 cells are a sub-population of pineal neurons lacking *foxd3* expression, we performed double immunohistochemistry for mCherry and the neuronal marker HuC in *agrp2*:mCherry larvae. This revealed AgRP2 cells to be HuC negative (Fig. 2c, Supplementary Movie S1). These experiments strongly suggested that AgRP2 cells are neither photoreceptors nor HuC-positive neurons. We then examined the possibility that pineal AgRP2 cells might be interstitial cells. Glial fibrillary acidic protein (GFAP) is a marker for pineal interstitial cells^33^,^41^. Double immunohistochemistry for GFAP and GFP in *agrp2*:EGFP larvae revealed different patterns of expression. This strongly suggested that AgRP2 cells do not express GFAP and are therefore not pineal interstitial cells (Fig. 2d, Supplementary Movie S1). Altogether, these results indicated that AgRP2 cells may represent a novel, uncharacterized pineal cell population.

### AgRP2 preoptic neurons project to the adenohypophysis

**Figure 3:**
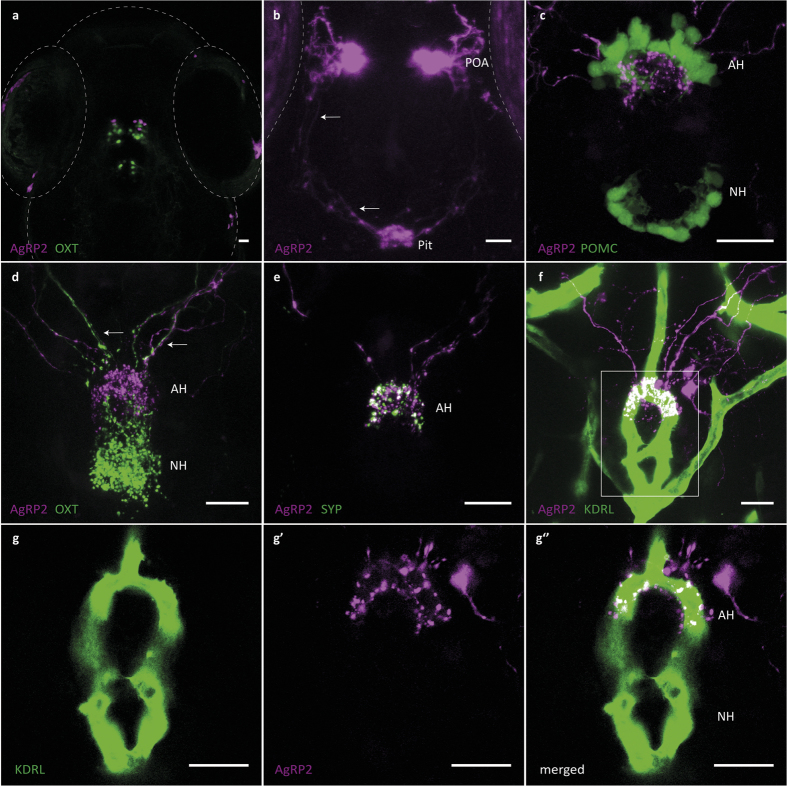
AgRP2 preoptic neurons project towards the pituitary and terminate at the pituitary vasculature. (**a**) A cross between *agrp2*:mCherry and Tg(*oxt*:EGFP)^wz01^ shows that AgRP2 neurons (magenta) are localized to the preoptic area, adjacent to oxytocin neurons (green), dorsal view of a 5-dpf larva head. (**b**) Higher-gain imaging of a 5-dpf *agrp2*:mCherry larva reveals projections of the AgRP2 preoptic neurons towards the pituitary (arrows). (**c**) Pituitary of a 12-dpf *agrp2*:mCherry, Tg(*pomc*:EGFP)^zf44^ larva, showing AgRP2 projections (magenta) terminating at the adenohypophysis, in close proximity to anterior POMC neurons (green). (**d**) Pituitary of a 12-dpf *agrp2*:mCherry, Tg(*oxt*:EGFP)^wz01^ larva, showing that projections from AgRP2 (magenta) and oxytocin (green) neurons share the same axonal tracts (arrows) but terminate at different locations within the pituitary: AgRP2 axons terminate at an anterior region whereas oxytocin axons terminate at a posterior region. (**e**) Pituitary of a 7-dpf *agrp2*:mCherry, Tg(UAS:SYP-EGFP)^biu5^ larva expressing synaptophysin (SYP)-EGFP fusion protein and mCherry in AgRP2 neurons. SYP-EGFP protein aggregates in pre-synaptic vesicles at the adenohypophysial AgRP2 terminals. (**f**) Z-stack projection of a 12-dpf *agrp2*:mCherry, Tg(*kdrl*:EGFP)^s843^ larval pituitary, showing that AgRP2 preoptic projections (magenta) form an interface with the pituitary vasculature (green). (**g**−**g”**) show higher magnification of (**f**). (**g**) A single focal plane of the pituitary vasculature. (**g’**) AgRP2 terminals form an arc along the pituitary artery. (**g”**) Merged image of (**g**) and (**g’**). The observed overlap between AgRP2 terminals and the pituitary artery demonstrates a neurovascular interface of AgRP2 terminals with the pituitary vasculature. Anterior to top. AH, adenohypophysis; NH, neurohypophysis; Pit, pituitary; POA, preoptic area. Scale bar, 20 μm.

We next investigated the other main *agrp2* expression domain, a small bilateral group of cells in the preoptic area (4−5 cells in each hemisphere, Fig. 3a). Confocal imaging of *agrp2*:mCherry revealed that these cells project towards the pituitary (Fig. 3b, Supplementary Movie S2). Examination of transgene expression in a cross between *agrp2*:mCherry and Tg(*oxt*:EGFP)^wz01^ fish^42^ showed that AgRP2-positive cells and oxytocin neurons are adjacent (Fig. 3a) and project towards the pituitary (Fig. 3d). The presence of axon-like processes and the sharing of axonal tracts (Fig. 3d) suggest that these cells are neurons. We therefore examined these AgRP2 axonal projections. A cross between *agrp2*:mCherry and Tg(*pomc*:EGFP)^zf44^ fish (which expresses EGFP in pituitary POMC cells only^43^) revealed that AgRP2 axons terminate near the anterior group of pituitary POMC cells (Fig. 3c), suggesting that the preoptic AgRP2 neurons project towards the adenohypophysis. This anatomical location was confirmed by comparing the terminations of AgRP2 and oxytocin axons in the pituitary: a clear segregation was observed between these terminals in the adenohypophysial and neurohypophysial domains, with AgRP2 fibres terminating in an anterior zone and oxytocin fibres in a posterior zone of the pituitary (Fig. 3d).

To verify that the AgRP2 adenohypophysial terminations are indeed axonal terminals, we crossed *agrp2*:mCherry fish with Tg(UAS:SYP-EGFP)^biu5^ fish. This UAS-Gal4 driven line expresses a synaptophysin-EGFP fusion protein that aggregates in pre-synaptic vesicles^44^,^45^. In the progeny of this cross, AgRP2 cell bodies and projections are labelled with mCherry, while pre-synaptic varicosities are labelled with EGFP. Examination of the larvae resulting from this cross revealed a field of EGFP-positive pre-synaptic boutons in the AgRP2 pituitary terminals (Fig. 3e). Neurovascular connections reminiscent of the tetrapod hypophysial portal system were recently described in the zebrafish adenohypophysis^46^. To examine the possibility of an AgRP2 neurovascular connection in the hypophysis, we crossed *agrp2*:mCherry with Tg(*kdrl*:EGFP)^S843^ fish (which expresses EGFP in vascular endothelial cells^47^). Examination of transgene expression in this cross showed that AgRP2 terminals are juxtaposed with vessels that have been designated as the hypophysial artery^42^ (Fig. 3f,g). AgRP2 projections towards the pituitary vasculature could be detected as early as 3 dpf; the complexity of the pituitary vasculature and the magnitude of AgRP2 innervation subsequently increased (described in Supplementary Fig. S2).

These results indicate that hypothalamic AgRP2 neurons are hypophysiotropic and probably terminate directly onto the hypophysial vasculature. Whether AgRP2 acts on adenohypophysial cells to induce hormonal secretion or is directly released into the circulation and has a neuroendocrine role in the periphery remains to be determined.

### AgRP1 projections and AgRP1-AgRP2 interactions

**Figure 4:**
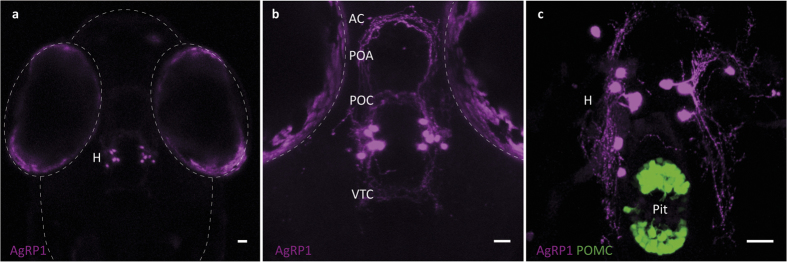
Anatomical organization of AgRP1 neurons. (**a**) Dorsal view of a 5-dpf *agrp1*:mCherry larva, showing AgRP1 somata localized at the ventral periventricular hypothalamus (H). (**b**) Higher magnification of the hypothalamus reveals AgRP1 projections towards the rostral, intermediate and dorsal hypothalamus, the preoptic area (POA), the anterior commissure (AC), the post-optic commissure (POC) and the ventral tegmental commissure (VTC). (**c**) Ventral view of a 8-dpf *agrp1*:mCherry; Tg(*pomc*:EGFP)^zf44^ larva shows that the AgRP1 neurons (magenta) do not project to the pituitary (Pit, green). Scale bar, 25 μm.

The zebrafish AgRP1 neuronal system has been previously described in a detailed immunohistochemical study^27^. In the present study we closely examined the generated *agrp1*:mCherry fish to determine whether it accurately represents the previously described distribution. These investigations revealed that AgRP1 neurons are located in the ventral periventricular hypothalamus (10−12 neurons in each hemisphere) and project axons towards the rostral, intermediate and dorsal hypothalamus, the preoptic area, the anterior commissure, the post-optic commissure, and the ventral tegmental commissure (Fig. 4a,b, Supplementary Movie S3), as previously described^27^. In contrast to the previous interpretation of immunohistochemical data^27^,^48^, however, the mCherry-expressing AgRP1 neurons in our study did not appear to project towards the pituitary. This was further validated in the double-transgenic larvae *agrp1*:mCherry, Tg(*pomc*:EGFP)^zf44^, which exhibited no AgRP1 projections or terminals at the pituitary territory (Fig. 4c, Supplementary Movie S4). AgRP2 projections towards the pituitary may have been previously mistaken for AgRP1 projections because the antibody used in those studies was shown to bind both AgRP1 and AgRP2^27^,^48^, and because *agrp2* expression was considered at that time to be restricted to the pineal.

**Figure 5:**
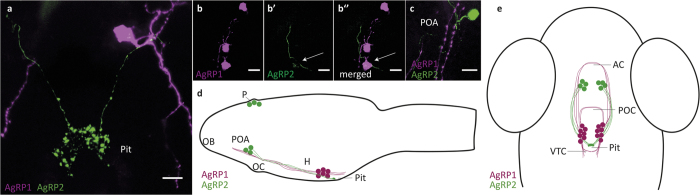
AgRP1 and AgRP2 projections and their interactions. Analysis of potential interactions between AgRP1 and AgRP2 neurons by crossing of *agrp1*:mCherry with *agrp2*:EGFP fish. (**a**) AgRP2 projections (green) towards the adenohypophysis pass alongside AgRP1 somata (magenta). (**b**−**b**”) AgRP2 projections potentially form ‘en passant’ synapses with AgRP1 somata. Arrow points to a swelling along the AgRP2 axon in (**b’**). (**b”**) Merged image of (**b**) and (**b’**), showing co-localization of AgRP2 axonal swelling (green) and AgRP1 soma (magenta). (**c**) In the pre-optic area, AgRP1 axons (magenta) pass close to AgRP2 somata (green) and share the same axonal tracks with AgRP2 axons. (**d**,**e**) Schematic diagrams showing lateral (**d**) and dorsal (**e**) views of the anatomical organization of AgRP1 and AgRP2 neuronal systems in 5-dpf larvae. AC, anterior commissure; H, hypothalamus; OB, olfactory bulb; OC, optic chiasm; P, pineal; Pit, pituitary; POA, pre-optic area; POC, post-optic commissure; VTC, ventral tegmental commissure. Scale bar, 10 μm.

To examine the possibility of a relationship between AgRP1 and AgRP2 neurons in the hypothalamus, we crossed *agrp1*:mCherry with *agrp2*:EGFP fish. Imaging of the resulting larvae revealed the presence of what appeared to be ‘en passant’ synapses of AgRP2 preoptic projections on AgRP1 somata. Thus, AgRP2 axons were in close proximity to AgRP1 cell bodies and appeared to innervate them while projecting to the pituitary (Fig. 5a,b). Reciprocal connectivity also appeared to occur, as the AgRP1 axonal projections towards the preoptic area innervated AgRP2 somata (Fig. 5c). There was also close apposition between hypothalamic AgRP1 and AgRP2 axons as they traversed the same tracts between the preoptic area and the ventral periventricular hypothalamus. This anatomical evidence suggested close association and possible physiological inter-regulation between AgRP1 and AgRP2 neurons (as shown schematically in Fig. 5d,e).

## Discussion

The ancestors of teleost fish, after diverging from the ancestors of tetrapods, underwent a whole-genome duplication event. Many of the resulting paralogous genes^22^,^23^, called ohnologues, quickly acquired mutations leading to one of a few possible outcomes: degeneration of one of the paralogous genes while the other retained its original function; partition of the pre-existing function (subfunctionalization); or emergence of new functions (neofunctionalization)^49^,^50^. Four genes belonging to the agouti family have been identified in fish, *agrp1, agrp2, agouti-signaling protein 1 (asip1*) and *asip2*. Their phylogenetic relationship is still unclear; specifically, whether *agrp1* and *agrp2* are duplicates or *agrp2* is a paralog of *asip1*, is a matter of debate^24^,^26^,^51^. The anatomical distribution of the AgRP1 and AgRP2 neurons and of their projections in the zebrafish brain suggests that both subfunctionalization and neofunctionalization may have occurred: in the preoptic area and the hypothalamus each of the two *agrp* genes may have retained partial roles of the ancestral *agrp*, while in the pineal gland *agrp2* has acquired a new function.

The pineal gland of non-mammalian vertebrates is photoreceptive, contains an intrinsic circadian oscillator, and influences daily rhythms and seasonal changes^33^,^52^,^53^. The role of the pineal gland in transducing photoperiodic information is particularly apparent in many teleost fish species, where it is located just underneath a thin, translucent area of the skull overlaid by less pigmented skin known as the pineal window, which facilitates the entry of light^52^. Three main types of cells have been characterized in the fish pineal gland: photoreceptor cells, projection neurons, and interstitial cells^33^,^34^,^35^,^36^. Data mining of pineal gland transcriptomes in zebrafish^28^,^29^,^54^ reveals that *agrp2* mRNA is among the most highly expressed transcripts in this tissue (data not shown). Remarkably, the pineal AgRP2 cells described here do not express known markers for pineal photoreceptor cells (AANAT2), pineal neurons (FoxD3 and HuC), or glia (GFAP), and therefore may represent a previously uncharacterized type of pineal cell. Expression of melanocortin receptors in the pineal gland (Supplementary Fig. S3) suggests that the AgRP2 peptide exerts intracellular communication within the pineal gland. The current identification of previously uncharacterized AgRP2 pineal cells possibly reflects neofunctionalization of the *agrp2* gene. The functional properties of these cells clearly warrant further thorough investigation.

Compared with the pineal AgRP2 cells, the preoptic AgRP2 neuronal system appears to have more characteristics in common with the ancestral AgRP neuronal system. The functional and anatomical properties of the AgRP neuronal network have been extensively studied in mammals^55^,^56^,^57^,^58^. AgRP terminals were shown to be widely spread throughout several hypothalamic structures as well as the amygdala, other forebrain regions and brainstem^55^,^56^. AgRP terminals were also found in the internal layer of the median eminence in rodents^56^,^59^, the infundibulum in rhesus monkeys^60^, and the infundibulum and median eminence in ducks^61^. Those findings suggest that AgRP may also act as a hypophysiotropic factor and thus exert neuroendocrine functions, a potential property of AgRP that has been largely overlooked. In the present study we show that zebrafish AgRP1 neurons project axons to multiple locations including rostral, intermediate and dorsal areas of the hypothalamus and the preoptic area, but do not project to the pituitary. AgRP2 preoptic neurons, on the other hand, project directly towards the pituitary, where they form a neurovascular interface with the hypophysial artery. This finding suggests that AgRP2 neurons are hypophysiotropic and may affect pituitary hormonal secretion. Expression of *agrp2* mRNA was previously detected by PCR in the brains of sea bass^20^, Atlantic salmon (*Salmo salar*)^18^ and pufferfish (*Takifugu rubripes*)^17^, suggesting that the preoptic AgRP2 system may not be specific to zebrafish. Thus, the present anatomical findings, together with previous studies in zebrafish, suggest this is an example of subfunctionalization, in which *agrp1* has retained its function as a central regulator of food consumption^27^,^62^, while *agrp2* has taken over the putative neuroendocrine role of *agrp* in the pituitary.

A possible neuroendocrine role for AgRP2 in zebrafish is in the regulation of background adaptation. Transient knock-down of AgRP2, using morpholino-modified oligonucleotides, prevented pigment aggregation when larvae were placed on a white background^30^. Since *agrp2* was thought to be expressed exclusively in the pineal, it was suggested that AgRP2 is expressed in pineal neurons that regulate pigmentation through MC1R signaling and activation of hypothalamic melanin-concentrating hormone neurons^30^. We now propose an alternative mechanism of action for AgRP2. The current findings of preoptic hypophysiotropic AgRP2 neurons, together with the absence of pineal AgRP2 projections to the hypothalamus, suggest that the effect of AgRP2 on background adaptation might be exerted directly via the synapses of AgRP2 neurons in the hypophysial vasculature. Possible mechanisms for the effect of hypophysiotropic AgRP2 on pigmentation are: a) release of AgRP2 into the general circulation, thus directly affecting MC1R signaling throughout the skin and allowing for rapid background adaption; and b) modulation of pituitary melanotrope cells by AgRP2. Pituitary α-MSH affects background adaption in teleost^3^, and zebrafish melanosomes disperse rapidly in response to α-MSH^63^. However, the lack of melanocortin receptor expression in the pituitary^64^ (and Supplementary Fig. S3) would suggest that such hypophysiotropic activity, if exists, is mediated by a different type of receptor. In both cases, knock-down of AgRP2^30^ would be expected to increase MC1R activation by α-MSH, leading to the dispersion of melanosomes and decreased camouflage capability.

Intriguingly, AgRP2 axonal projections coursing towards the pituitary form what appear to be en passant synapses with AgRP1 cell bodies, suggesting that AgRP2 neurons can directly modulate AgRP1 neuronal activity. These connections appear to be reciprocal, with AgRP1 projections towards the preoptic area overlapping with AgRP2 projections and cell bodies. This suggests a possible mutual modulation, in which AgRP2 neurons modulate feeding via AgRP1 neurons and AgRP1 neurons modulate background adaptation via AgRP2 neurons. Whilst it is certainly highly speculative to suggest, it is possible that this mutual modulation reflects the relationship between food foraging and predation avoidance. Thus, background conditions that increase the likelihood of predation might simultaneously stimulate the induction of camouflage and suppression of appetite.

In this study we generated genetically modified zebrafish lines, thereby enabling anatomical and functional investigations of the AgRP systems. We propose that whereas zebrafish hypothalamic AgRP1 neurons function as central regulators of feeding, preoptic AgRP2 neurons have a neuroendocrine function, possibly related to pigmentation and camouflage. We showed that the two types of AgRP neurons may communicate and modulate one another. Pineal *agrp2* expression appears to be a unique feature of teleosts, but its importance for pineal function remains unclear. The Gal4 transgenic lines generated here will facilitate future investigations of the AgRP1 and AgRP2 systems through the use of a variety of UAS transgenic lines. Approaches such as neuronal ablation and silencing, neuronal activation, and the monitoring of neuronal activity in these systems should now be feasible. Overall, these transgenic fish provide exciting new models for studying the neuronal mechanisms regulating food consumption and feeding behavior as well as the neuroendocrine roles of hypothalamic AgRP2 and the novel functions of pineal AgRP2.

## Materials and Methods

### Ethics statement

All procedures were approved by the Tel Aviv University Animal Care Committee and conducted in accordance with the requirements of the Council for Experimentation on Animal Subjects, Ministry of Health, Israel.

### Fish maintenance

Adult zebrafish were raised in a recirculation water system under 12 h light:12 h dark cycles at 28 °C and fed twice a day. Embryos were generated by natural mating, placed in 10 cm Petri dishes with egg water containing methylene blue (0.3 ppm), and raised in a light-controlled incubator at 28 °C (light intensity, 12 W/m^2^). To prevent pigmentation for ISH and immunostaining analysis, the fish water was supplemented with 0.2 mM phenylthiourea from 1-dpf onward.

### BAC recombineering and transgenesis

BAC clones CH73-27D24 and CH73-352F23, containing AgRP1 and AgRP2 coding sequences, respectively, were obtained from the BACPAC Resources Center. The BAC plasmids were introduced by electroporation into *Escherichia coli* SW102 strain (kindly provided by Donald Court), which contains heat-inducible recombinase functions^65^. Tol2 and reporter gene cassettes (GFP or Gal4-VP16, kindly provided by Maximiliano Suster) were recombineered into the BAC clones according to established protocol^31^,^66^ (Supplementary Fig. S1). Reporter gene cassettes were amplified by PCR together with 50 bp homology arms of *agrp1* or *agrp2* 5′ untranslated region and first exon, with consequent recombination of the reporter gene after the AgRP1- or AgRP2-translation initiation site (Supplementary Table S1). Tol2 was amplified and recombineered as described^31^. Recombineered BAC clones were purified using HiPure Plasmid Midiprep Kit (Invitrogen), according to the manufacturer’s instructions, and co-injected with Tol2 transposase RNA into single-cell embryos as described^31^. Embryos were raised and their progeny screened for positive germline transmission.

### Whole-mount *in-situ* hybridization

*agrp2* mRNA sequence (reference sequence NM_001271291.1, bp 41–544) and *agrp1* mRNA sequence (reference sequence NM_001328012.1, bp 23–584) were cloned into pGEM-T Easy (Promega), linearized, and used as template for *in-vitro* synthesis of digoxigenin (DIG)-labelled anti-sense riboprobes (DIG RNA labelling kit, Roche). Wild-type 6-dpf larvae were fixed in 4% paraformaldehyde and stored in 100% methanol. Whole-mount ISH analysis was carried out according to an established protocol^67^. For image analysis, the ISH-stained larvae were placed in 70% glycerol and photographed with a dissecting microscope (SZX12, Olympus) equipped with a digital camera (DP70, Olympus). The ISH was repeated three times, each time with 15−30 larvae for each gene.

### Immunostaining

Transgenic 5-dpf larvae were fixed in 4% paraformaldehyde and stored in 100% methanol. The immunostaining protocol was performed as previously described^68^. The primary antibodies used were mouse anti-GFP (1:100, MBL International Cat# M048-3); rabbit anti-GFAP (1:80, Sigma-Aldrich Cat# G9269); mouse anti-HuC/HuD (15 μg/ml, Molecular Probes Cat# A-21271), and rabbit anti-RFP (1:1000, MBL International Cat# PM005). Secondary antibodies used were Alexa Fluor 488 donkey anti-mouse IgG (1:100, Jackson ImmunoResearch Cat# 715-545-150) and Alexa Fluor 594 donkey anti-rabbit IgG (1:100, Jackson ImmunoResearch Cat# 711-585-152). The immunostaining was repeated twice with 8−10 larvae each time.

### Confocal microscopy imaging

Transgenic 3−12-dpf larvae were anesthetized and placed in low melting agarose for dorsal imaging. For lateral and ventral imaging the larvae were fixed in 4% paraformaldehyde, washed in PBS, and their eyes and jaws were dissected. At least five larvae of each genotype combination were analyzed. All images were obtained using a Leica TCS SP8 confocal laser scanning microscope equipped with Leica LAS AF image acquisition software.

## Additional Information

**How to cite this article:** Shainer, I. *et al*. Novel hypophysiotropic AgRP2 neurons and pineal cells revealed by BAC transgenesis in zebrafish. *Sci. Rep.* **7**, 44777; doi: 10.1038/srep44777 (2017).

**Publisher's note:** Springer Nature remains neutral with regard to jurisdictional claims in published maps and institutional affiliations.

## Supplementary Material

Supplementary Movie S1

Supplementary Movie S2

Supplementary Movie S3

Supplementary Movie S4

Supplementary Information
