# Supplementary material for: Novel hypophysiotropic AgRP2 neurons and pineal cells revealed by BAC transgenesis in zebrafish
Source: Sci Rep. 2017 Mar 20;7:44777. doi: 10.1038/srep44777 (PMC5357965; doi:10.1038/srep44777)
Supplement: Supplementary Information [file srep44777-s5.pdf]

Novel hypophysiotropic AgRP2 neurons and pineal cells revealed by BAC transgenesis in zebrafish

Inbal Shainer[1], Adi Buchshtab[1], Thomas A. Hawkins[3], Stephen W. Wilson[3], Roger D. Cone[4] and Yoav Gothilf[1,2*]

[1]Department of Neurobiology, George S. Wise Faculty of Life Sciences, Tel-Aviv University, Tel-Aviv, Israel.

[2]Sagol School of Neuroscience, Tel-Aviv University, Tel-Aviv, Israel.

[3]The Department of Cell and Developmental Biology, Faculty of Life Sciences, University College London, London, UK.

[4]Life Sciences Institute, University of Michigan, Ann Arbor, MI, USA.

* Correspondence should be addressed to Y.G. (email yoavgothilf@gmail.com)

Supplementary Methods

Analysis of melanocortin receptors RNA expression

Adult fish were scarified and the following tissues were carefully removed for RNA extraction: a female pituitary, a male pituitary, a pool of 6 pineal glands, and a pool of 4 brains. Total RNA was extracted using RNeasy Lipid Tissue Mini Kit (QIAGEN) according to the manufacturer's instructions. To remove genomic DNA, On-Column DNase digestion was performed with an RNase-Free DNase Set (QIAGEN). Up to 1 μg of RNA was used for cDNA preparation using the qScript cDNA Synthesis Kit (Quantabio) according to the manufacturer's instructions.

The cDNA samples were PCR-amplified by a ReddyMix PCR Master Mix (Thermo-Fisher Scientific) with specific primers: *gh* primers, 5'-accaaccttcaatcaagaacg-3' and 5'-tggtcttagatttgcagaaaagg-3'; *mc1r* primers, 5'-catcacttcagcatgaaacaca-3' and 5'- tgggacaggtgagaattaggat-3'; *mc3r* primers, 5'- agcacttctttgcctcctaatg-3' and 5'- ggtcaatcacagagttgcacat-3'; *mc4r* primers, 5'-gagaacatccttgtgattgcag-3' and 5'-cagagatgcaagtccataccag-3'; and *agrp2* primers, 5'-cccctttatcaggtcggaaa-3' and 5'-gcgcaatcaacgctgaagta-3'. PCR was performed with initial denaturation at 95 °C for 5 min, followed by 40 cycles of 94°C for 30 s, 56°C for 30 s, 72°C for 60 s, and final elongation at 72°C for 5 min.

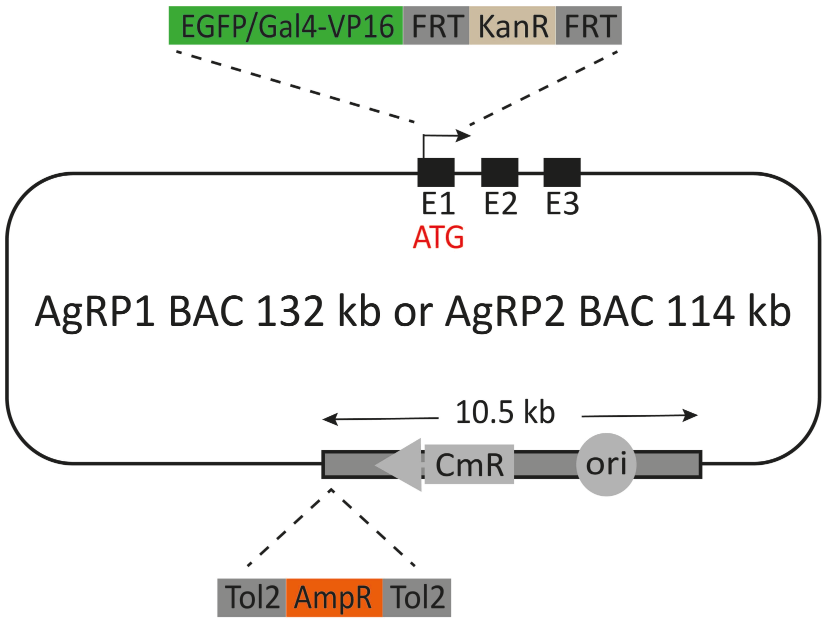

Supplementary Figure S1. Schematic representation of the BAC clones used for transgenesis. Clones of 132 kb and 114 kb clones were used for transgenesis of *agrp1* and *agrp2*, respectively. The clones were recombineered to include Tol2 sequences in the plasmid backbone and Gal4-VP16 or EGFP immediately after the AgRP1 and AgRP2 translation initiation sites.

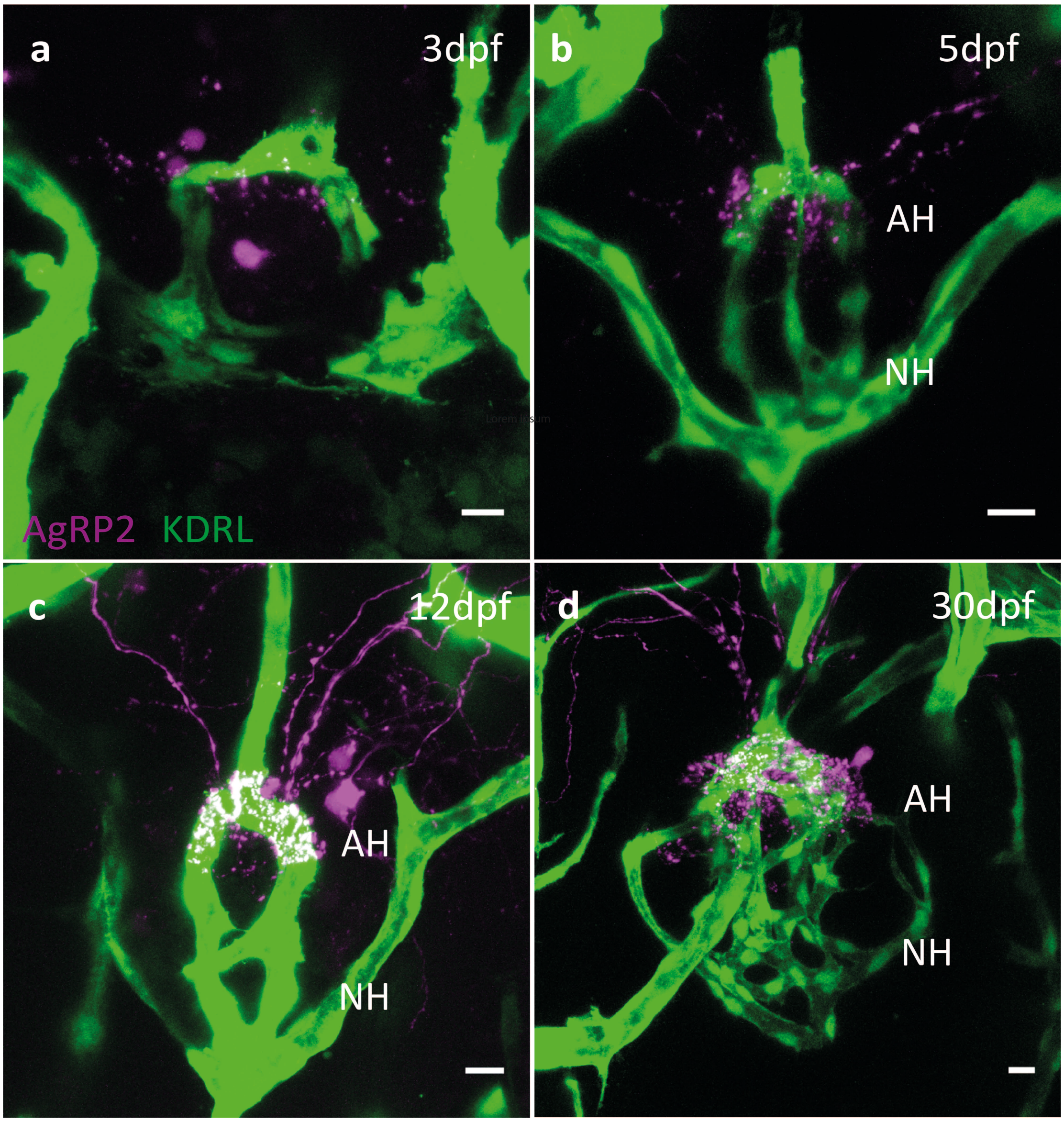

Supplementary Figure S2. Development of AgRP2 projections (magenta) towards the pituitary vasculature (green). (**a**) AgRP2 preoptic neuronal projection towards the pituitary vasculature is detected as early as 3 dpf. (**b**) At 5 dpf, as the pituitary vasculature continues to develop; more AgRP2 projections can be detected. (**c**) At 12 dpf, a clear interface can be detected between AgRP2 and the pituitary vasculature. (d) At 30 dpf, complexity of the pituitary vasculature increases together with increased number of AgRP2 terminals.

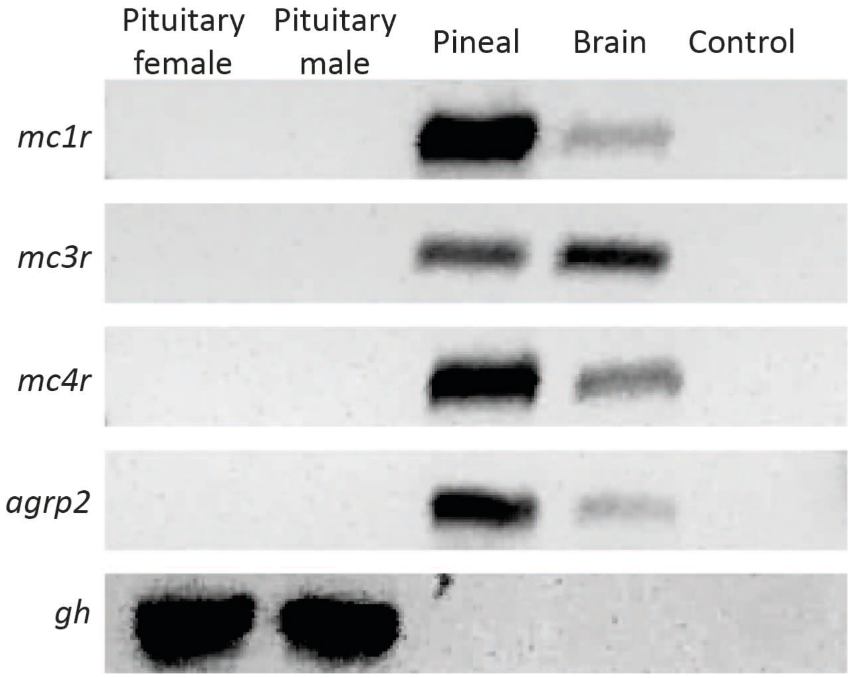

Supplementary Figure S3. Analysis of spatial expression pattern of melanocortin receptors. RT-PCR analysis of melanocortin-receptor expression shows that *mc1r*, *mc3r* and *mc4r* mRNAs are expressed in the zebrafish brain and pineal, but not in the pituitary. Growth hormone (*gh*) serves as a positive control for the pituitary cDNA, and *agrp2* as a positive control for the brain and the pineal cDNA.

Primer	Sequence
*agrp2*_GFP_fw	gaataaggagactcacttcataaaaactcccctttatcaggtcggaaaatgGTGAGCAAGGGCGAGGAGCTGTTC
*agrp2*_Gal4-VP16_fw	gaataaggagactcacttcataaaaactcccctttatcaggtcggaaaatgGTGAAGCTACTGTCTTCTATCGAAC
*agrp2*_frt-kan_rev	ctactgtgaagaacaagcagatgaagagcacaaatcctttcagcaccgcCCGCGTGTAGGCTGGAGCTGCTTC
*agrp1*_Gal4-VP16_fw	aaaagtgtggctaaagtttatctcttttttgagtctgagtgattatgatgGTGAAGCTACTGTCTTCTATCGAAC
*agrp1*_frt-kan_rev	agatgccatcaccacaacattcaccaaaaaccagccgaagattactgtCCGCGTGTAGGCTGGAGCTGCTTC

Supplementary Table S1. Primers used to amplify reporter genes with *agrp2* or *agrp1* homology arms.

Supplementary Movie S1. AgRP2 is expressed in uncharacterized pineal cells. Confocal images of AgRP2 cells with cells expressing AANAT2, FoxD3, HuC or GFAP revealed that AgRP2 cells are novel pineal cells.

Supplementary Movie S2. Preoptic AgRP2 neurons project towards the pituitary. Confocal 3D imaging**.**

Supplementary Movie S3. 3D imaging of AgRP1 neurons and projections.

Supplementary Movie S4. 3D imaging of AgRP1 neurons and projections and pituitary POMC cells. AgRP1 neurons (grey) do not project towards the pituitary, represented by POMC cell (green).
